# NanoSSL: attention mechanism-based self-supervised learning method for protein identification using nanopores

**DOI:** 10.1093/bioinformatics/btaf657

**Published:** 2025-12-05

**Authors:** Yong Xie, Jindong Li, Ziyan Zhang, Bin Meng, Shuaijian Dai, Yuchen Zhou, Eamonn Kennedy, Niandong Jiao, Haobin Chen, Zhuxin Dong

**Affiliations:** Department of Biomedical Engineering, Xiangya School of Basic Medical Sciences, Central South University, Changsha, Hunan 410013, China; Department of Biomedical Engineering, Xiangya School of Basic Medical Sciences, Central South University, Changsha, Hunan 410013, China; Department of Biomedical Engineering, Xiangya School of Basic Medical Sciences, Central South University, Changsha, Hunan 410013, China; Department of Biomedical Engineering, Xiangya School of Basic Medical Sciences, Central South University, Changsha, Hunan 410013, China; Department of Biomedical Engineering, Xiangya School of Basic Medical Sciences, Central South University, Changsha, Hunan 410013, China; Xiangya School of Medicine, Central South University, Changsha, Hunan 410013, China; Division of Epidemiology, Internal Medicine, University of Utah, Salt Lake City, UT 84112, United States; State Key Laboratory of Robotics, Shenyang Institute of Automation, Chinese Academy of Sciences, Shenyang, Liaoning 110169, China; Department of Biomedical Engineering, Xiangya School of Basic Medical Sciences, Central South University, Changsha, Hunan 410013, China; Furong Laboratory, Changsha, Hunan 410000, China; National Engineering Research Center of Personalized Diagnostic and Therapeutic, Changsha, Hunan 410000, China; Department of Biomedical Engineering, Xiangya School of Basic Medical Sciences, Central South University, Changsha, Hunan 410013, China; Furong Laboratory, Changsha, Hunan 410000, China; National Engineering Research Center of Personalized Diagnostic and Therapeutic, Changsha, Hunan 410000, China

## Abstract

**Motivation:**

Nanopores are cutting-edge interdisciplinary tools that can analyze biomolecules at the single-molecule level for many applications, e.g. DNA sequencing. Efforts are underway to extend nanopores to proteomics, including the development of machine learning algorithms for protein sequencing and identification. However, single-molecule data are intrinsically noisy and hard to process. Moreover, the development and performance of machine learning for nanopore is jeopardized by data scarcity. Self-supervised learning is an emerging method that may yield advantages in nanopore scenarios.

**Results:**

We propose and experimentally validate Nanopore analysis using Self-Supervised Learning (NanoSSL), a generative self-supervised learning framework based on attention mechanisms for the identification of protein signals from nanopores. Leveraging a two-step approach consisting of self-supervised pre-training and supervised fine-tuning, NanoSSL learns useful feature representations from empirical data to facilitate downstream classification tasks. Inspired by the concept of fragmentation in conventional protein sequencing technologies, during pretraining each translocation event is split into multiple non-overlapping fragments of equal size, some of which are randomly masked and reconstructed using a masked autoencoder. Learning the feature representations of the reconstructed nanopore events facilitates molecular identification in fine-tuning. In this study, we retested a publicly available nanopore multiplexed protein sensing dataset for model iteration, and subsequently measured Alzheimer’s disease biomarker Aβ_1-42_ using homemade solid-state nanopores. Empirical results indicated NanoSSL achieved an unprecedented performance across four metrics: accuracy, precision, recall, and F1 score, when classifying two mutated Aβ_1-42_, E22G and G37R. The self-supervised learning and attention mechanism were verified as the source of performance gains.

**Availability and implementation:**

The main program is available at https://doi.org/10.5281/zenodo.17172822.

## 1 Introduction

In recent years, nanopore platforms have been widely recognized as viable third-generation DNA sequencing technologies, providing high throughput, low cost, and long read length gene sequencing (Manrao *et al.* 2012, Bayley 2015). Nanopore technologies are gradually extending to the field of proteomics (Afshar Bakshloo *et al.* 2022, Mayer *et al.* 2022, Ying *et al.* 2022, Dorey and Howorka 2024). By analyzing the blocking current caused by single molecules translocating through nanopores, subtle information about molecular characteristics can be obtained. However, generating and analyzing nanopore protein sequencing data faces specific challenges (Si and Aksimentiev 2017). Compared to the combination of four bases in a DNA or RNA strands, proteins have a much more complex and smaller chemical structure (LaPelusa and Kaushik 2024). The difficulty of analyzing nanopore protein translocation events at the single-molecule level significantly increases due to the presence of up to 20 types of proteinogenic amino acids of varying charge, hydrophilicity, and hydrophobicity. Moreover, protein sequencing with nanopores is a new technology with limited publicly available data for algorithm development or validation.

Machine learning methods have been widely adopted in signal recognition and data mining of molecular measurements from nanopores (Taniguchi *et al.* 2021, Wang *et al.* 2022, Motone *et al.* 2024, Yin *et al.* 2024). Unfortunately, traditional machine learning methods typically rely on manual feature selection or labelling, which requires experienced specialists to spend a lot of time filtering features, leading to low efficiency, small sample size, and slow adoption. For example, a recent study identified signals from different RNA molecules by applying various classical machine learning algorithms (Liu *et al.* 2022). This method performed well, but required manual selection of 11 features, limiting the generalizability of the work beyond specific data scenarios.

In the meantime, deep learning has achieved remarkable progress in many fields such as computer vision (Voulodimos *et al.* 2018, Esteva *et al.* 2019), natural language processing (Young *et al.* 2018, Li *et al.* 2021, Otter *et al.* 2021), and speech recognition (Yoo *et al.* 2022), and has shown great potential in other fields such as biomedicine (Stahlschmidt *et al.* 2022). As an end-to-end analysis tool, deep learning methods simplify the step of complex feature selection, and offer advantages for analyzing nanopore data (Zeng *et al.* 2019). A convolutional neural network (CNN)-based model called QuipuNet (Misiunas *et al.* 2018) has been developed for the dataset of multiplexed single-molecule protein sensing, which increased the amount of useful nanopore signal events by five-fold, and classified eight molecular ‘barcodes’ and corresponding proteins with an unprecedented accuracy. Another work utilized the combination of CNN and Long Short-Term Memory (LSTM) to identify the fluctuation patterns of different peptides translocating in solid-state nanopores, and consequently an important biomarker, beta-amyloid (Aβ_1-42_), and its scrambled version were successfully classified at the single-molecule level (Chen *et al.* 2024).

As new deep learning paradigms emerge, they are likely to continue to improve the quality of nanopore data analysis, thereby expanding the application scope of nanopore proteomics. However, deep learning nanopore studies to date are typically tasked to solve very specific problems, such as methylation recognition, target molecule binding recognition, etc. while the need for generalization is ignored. As a result, the utility of deep learning for nanopore applications remains limited, in part due to limited experimental data availability. Addressing these issues demands new approaches that can take advantage of unlabeled data. Self-supervised learning does not require annotated data (Ericsson *et al.* 2022, Rafiei *et al.* 2024), and allows models to merely utilize the structure or intrinsic relationships of the data to discover potential feature representations in the data. Thus, it could become a solution to nanopore signal recognition problems.

In this study, we propose our deep learning method named NanoSSL (**Nano**pore analysis using **S**elf-**S**upervised **L**earning). NanoSSL’s main contributions include:

1. A generative self-supervised learning strategy that learns useful feature representations from complex nanopore protein single-molecule data. By aggregating specified adjacent sampling points in each nanopore event into localized subsequences, signal patterns become perceptible, which is found to be beneficial for pretraining.
2. An asymmetric autoencoder architecture, in which autoencoders are neural networks, can compress data into latent representations with minimal information loss. It contains a masked autoencoder-style encoder to process visible and masked portions of the input separately, and a decoder that processes the entire input in parallel. This design promotes computational efficiency and minimizes the gap between pretraining and fine-tuning stages introduced by masking. A multi-head attention mechanism is incorporated to improve identification.
3. Extensive testing on both public and self-produced nanopore datasets to validate the performance of NanoSSL. NanoSSL consistently improved classification performance across data sources, analytes, and nanopore types tested. The experimental results demonstrate the effectiveness of NanoSSL’s combined self-supervised learning, autoencoder, and attention mechanism architecture.

## 2 Materials and methods

### 2.1 Platform

#### 2.1.1 Framework of NanoSSL

**Figure 1.**
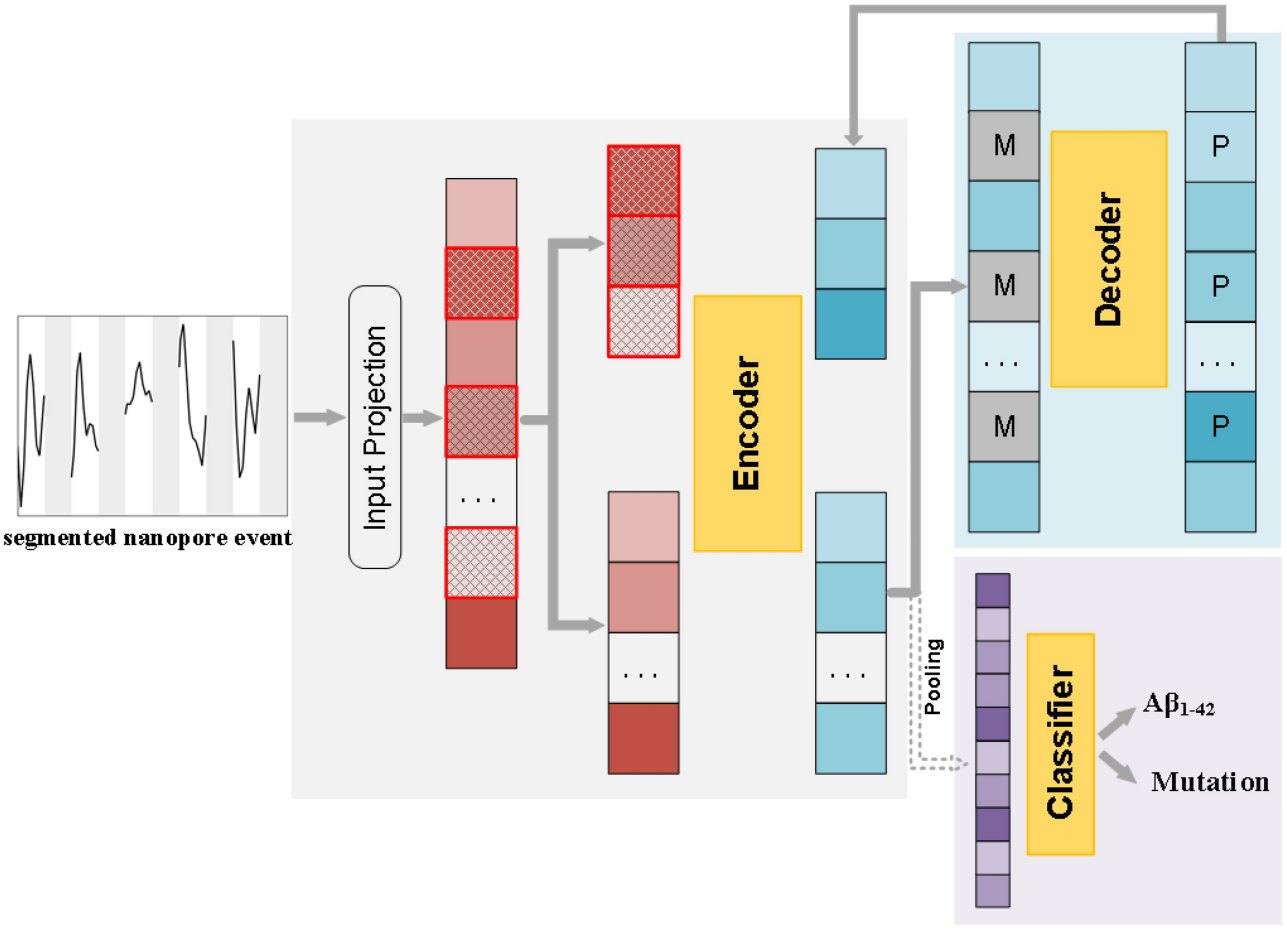
An overview of NanoSSL framework.

NanoSSL combines an attention mechanism and autoencoder architecture for representation learning of nanopore data at the single-molecule level to improve protein classification performance. As illustrated in Fig. 1, NanoSSL utilizes a two-step approach, consisting of self-supervised pretraining and supervised fine-tuning, to learn the most useful feature representations from complex nanopore signals without manual input, which is beneficial for downstream classification tasks. The pretraining stage completes the task of data mask-reconstruction through a generative self-supervised learning strategy. The fine-tuning stage optimizes the classification of nanopore signals using supervised learning.

#### 2.1.2 Self-supervised pre-training strategy

**Figure 2.**
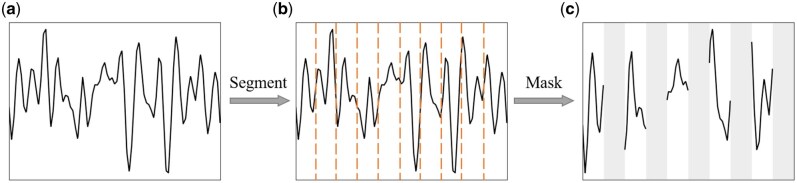
Illustration of nanopore current blockade signal processing. (a) One typical current blockade during a single denatured protein molecule translocating through a nanopore. (b) Segmentation of the temporal signal into multiple non-overlapping subsequences at the average amino acid periodicity. (c) Application of the mask.

The pretraining phase is designed to complete the task of data mask-reconstruction through an autoencoder architecture. First, each original nanopore current blockade with a length of sampling point *T* is defined as $S=\left.{\{s}_{1},s_{2},\;\ldots ,s_{T}\}\right.\in R^{T\times 1}$ (Fig. 2a). Inspired by similar approaches in protein fragmentation and segmentation in mass spectrometry imaging (Dosovitskiy *et al.* 2021, Nie *et al.* 2023), the nanopore signal *S* is segmented into *L* non-overlapping subsequences $P=\left.{\{p}_{1},p_{2},\;\ldots ,p_{L}\}\right.$. *L* can be determined by the number of amino acids constituting the protein and the spatial resolution offered by the nanopores. The home-made ultrathin subnanopores are capable of reading individual amino acids because the pore constriction can only accommodate one acid at a time. When a peptide chain manages to translocate through the pore at a constant velocity, one subsequence can approximate the observed signal from a corresponding amino acid (Chen *et al.* 2024). The mean number of residues estimated across all events is consistent with the length of the protein (Kennedy *et al.* 2016). Therefore, *L* is set to be 42 for processing the self-produced Aβ_1-42_ sequencing data as well as the public multiplexed protein sensing data of which we have not yet been inspired on the segmentation (Fig. 2b). These pointwise convergent subsequences as input leverages temporal context to extract robust features through noise averaging and local pattern integration (Jellema *et al.* 2010, Hu *et al.* 2021, Wang *et al.* 2022). Segmentation also reduces the length of the input data which improves the computational efficiency for the attention mechanism. A random masking strategy is applied to P with a mask ratio of r so that P is divided into visible $P_{v}$ and masked $P_{m}$ subsequences (Fig. 2c). Prior to the encoder, the input projection module is employed to linearly map the fragmented nanopore data P to a continuous feature space $X=\{X_{v},X_{m}\}\in R^{L\times d}$ with constant dimension, where d is the dimension of each element in the encoder (Section 3.1, available as supplementary data at *Bioinformatics* online).

Subsequently, the nanopore data are input into a masked autoencoder, which can implement both encoding and decoding based on multi-head attention mechanism (Section 3.2, available as supplementary data at *Bioinformatics* online). 8 layers of attention mechanism are used for both the encoder and the decoder. The encoder processes the masked input $X_{m}$ and the visible inputs$\;X_{v}$ separately, while the decoder simultaneously processes the entire input (Sections 3.3 and 3.4, available as supplementary data at *Bioinformatics* online). The masked-positional output vector $H_{m}^{8}$ of the encoder is considered as the target representation, while the masked-positional output vector $F_{m}^{8}$ of the decoder is considered as the predictive representation. Pre-training phase aligns the predicted representation with the target representation. The mean square error (MSE) function is used as the loss function for model assessment and it can be described by Equation (1), where *N* is the number of masked subsequences:


(1) MSE=1N∑i=1N(Fmi-Hmi)2

Together with the encoder’s processing strategy, this subsequence segmentation mechanism improves the pretraining efficiency (Table 1, available as supplementary data at *Bioinformatics* online), streamlining feature learning from local signals while reducing redundant computation when reconstructing masked regions.

**Table 1.** Dataset composition of Aβ_1-42_.

Protein	Samples	Number of nanopores
Native Aβ_1-42_	2395	5
E22G Aβ_1-42_	1963	4
G37R Aβ_1-42_	4931	3

#### 2.1.3 Supervised fine-tuning strategy

In the fine-tuning stage, the encoder deals with whole nanopore events. The input projection module and the encoder obtained from the pre-training stage are loaded to initialize the fine-tuning. According to each whole nanopore signal sequence P, the model generates a signal embedding $H^{8}$. Such signal embedding could be simplified through global average pooling operation, $H^{8}\in R^{L\times d}\to \;z\in R^{d}$.

After the encoder, nanopore signals associated with desired proteins be identified at the single-molecule level through an additional classifier (Section 3.5, available as supplementary data at *Bioinformatics* online). The cross-entropy (CE) loss function is selected as the loss function in this task. First, the output ${\hat{y}}_{c}$ of the classification head is converted into a probability distribution $p_{c}$ through the softmax operation as described in Equation (2), and then the CE between $p_{c}$ and the true category label $y_{c}$ of the nanopore data is calculated by Equation (3):


(2) pc=softmax⁡(y^c)=ey^c∑j=1Mey^j

(3) CE=-∑c=1Myclog⁡(pc)

### 2.2 Data

#### 2.2.1 Multiplexed single-molecule protein sensing data

The Oxford Nanopore Technologies (ONT) multiplex single-molecule protein sensing dataset originates from the work of Bell and Keyser (2016), which established a solid-state nanopore platform for multiplexed protein detection using DNA nanostructures. Further work provided a systematic deep learning analysis method based on this platform (Misiunas *et al.* 2018). The dataset consisted of ionic current signals in continuous time series. It was acquired by solid-state nanopores with a potential bias at 600 mV and intended for two progressive classification tasks. The first task was barcode identification, which was an 8-class classification problem aimed at distinguishing 8 DNA nanostructures with different binary barcodes from “000” to “111.” The encoding mechanism relied on the presence or absence of “hairpin loop” structures at known positions on the DNA carrier that produced characteristic current signals during translocation. The data comprised 52 525 training events and 3464 test events, and each event was pre-labelled with a specified barcode according to its signal feature. The second task was protein binding detection. This posed a binary classification problem to determine whether or not the target protein (IgG antibody) was bound to a predefined site on the DNA carrier. This specific binding was achieved via antigens that were conjugated to the DNA, and the DNA-protein complex generated translocation signals distinct from bare DNA carriers. This data comprised 17 147 binding events and 38 742 non-binding events (training and test sets combined), labelled as “Bound” or “Unbound.” Since all events had less than 700 data points, a 700-element vector was produced as the input, with padding around the events. In order to ensure the consistency between the evaluations, the same data processing method in the previous work was used in this study (Tables 2 and 3, available as supplementary data at *Bioinformatics* online).

**Table 2.** Classification results of antigen antibody barcode.^a^

Methods	Accuracy	Precision	Recall	F1 score
Bell & Keyser	N/A	0.937	0.182	0.305
Human	N/A	**0.978**	0.440	0.607
Transformer	0.919	0.923	0.914	0.919
SimCLR	0.866	0.844	0.801	0.810
QuipuNet	0.946	0.946	0.946	0.946
S2Snet	0.948	0.946	0.950	0.948
NanoSSL	**0.951**	0.950	**0.952**	**0.951**

a Best performances are bolded in the table.

**Table 3.** Classification results of target protein binding.^a^

Methods	Accuracy	Precision	Recall	F1 score
Bell & Keyser	N/A	0.940	0.192	0.319
Human	N/A	0.931	0.405	0.564
Transformer	0.935	0.942	0.878	0.909
SimCLR	0.964	**0.984**	0.918	0.950
QuipuNet	0.971	0.971	**0.971**	0.971
NanoSSL	**0.982**	**0.984**	0.969	**0.976**

a Best performances are bolded in the table.

#### 2.2.2 Solid-state nanopore protein sequencing data

This study focuses on the nanopore data from a subtype of beta-amyloid protein (Native Aβ_1-42_) related to Alzheimer’s disease and its two important mutants: E22G Aβ_1-42_, where glycine replaced glutamic acid at position 22, and G37R Aβ_1-42_, where arginine replaced glycine at position 37. The related amino acid sequences are listed in Table 4, available as supplementary data at *Bioinformatics* online. The protein monomers were denatured first by heating with surfactant and reducing agent. Then, the polypeptide chains were uniformly coated by the surfactant anions and driven through the nanopores by electrophoresis. The nanopores were e-beam sputtered in 5-nm-thick inorganic membranes with diameter less than 1 nm. Due to the steric constrain at the pore constriction, the anion shell was peeled off so that the amino acids were sensed one by one along the primary structure (Kennedy *et al.* 2016, Dong *et al.* 2017, Rigo *et al.* 2019, Chen *et al.* 2024). Each dataset of each protein type is sourced from multiple nanopores, consisting of thousands of current blockade samples (Table 1), and all the samples are normalized to 500 points using linear interpolation. These datasets are evenly divided into 5 parts to carry out five-fold cross validation and we take the average as the final result.

**Table 4.** Classification results between Aβ_1-42_ and its two mutants.^a^

Methods	E22G		G37R	
	ACC	F1-score	ACC	F1-score
SVM	0.865	0.859	0.882	0.907
RF	0.805	0.811	0.816	0.867
XGBoost	0.803	0.803	0.835	0.877
CNN	0.844	0.843	0.901	0.908
LSTM	0.821	0.819	0.895	0.893
Transformer	0.838	0.831	0.865	0.905
SimCLR	0.844	0.844	0.819	0.904
TandemMod	0.848	0.851	0.915	0.938
NanoSSL-ZeroTrain	0.830	0.846	0.893	0.915
NanoSSL	**0.895**	**0.887**	**0.925**	**0.945**

a Best performances are bolded in the table.

### 2.3 Experimental setup

In pretraining, the dimension size of the embeddings $d,\;d_{k},d_{v}$ in the attention module is set to 64. The mask ratio r is set to 0.6, and the length of the subsequence is set to 12. In the autoencoder framework, the layer number in the multi-head attention module of the encoder and decoder is set to 8, the number of heads, h, in each layer is set to 4, and the dropout rate $p_{1}$ in the feedforward network is set to 0.2. After the model is trained in 300 epochs, the Adam optimizer (Kingma and Ba 2015) is adopted as the default optimizer for all the deep learning methods in this study, with a learning rate set to 10^−3^ and a batch size set to 128.

In fine-tuning, the same hyperparameter settings as in the pre-training phase are retained. In addition, the output dimension $d_{c}$ of the first layer of the classifier is set to 256, and the dropout rate $p_{2}$ in the classifier is set to 0.2. The model is fine-tuned for another 300 epochs using the AdamW optimizer (Loshchilov and Hutter 2019), with an initial learning rate set to 10^−3^ and a weight decay coefficient set to 10^−4^. The learning rate is gradually reduced to 1% of the initial learning rate throughout the entire training cycle by adopting the cosine annealing learning rate scheduling strategy. To compare, a simplified version of NanoSSL equipped only with supervised learning was introduced, called NanoSSL-ZeroTrain, of which the experimental parameters remained consistent with the fine-tuning phase of NanoSSL.

The NanoSSL model was implemented by using the deep learning framework PyTorch (v1.13.1). All computational experiments were conducted on a workstation equipped with a 32 core CPU (Intel Xeon Platinum 8336C), 128GB of memory, and Nvidia GeForce GTX 4090 GPU × 3.

## 3 Results and discussion

### 3.1 Classification of public nanopore data

#### 3.1.1 Identification of antigen-antibody barcode

NanoSSL is initially applied to a publicly available ONT multiplex protein sensing dataset for recognizing the binding barcodes and target proteins separately. One important indicator is accuracy, which gives the proportion of correctly identified events. The F1 Score is another one which calculates the harmonic average of precision and recall. The definition of accuracy and F1 score is given in Section 4, available as supplementary data at *Bioinformatics* online. The original literature (Bell and Keyser 2016) presented a statistical analysis method referred to as Bell&Keyser and a human expert recognition methods referred to as Human. A follow-up (Misiunas *et al.* 2018) reported a recognition accuracy of 0.946 for 8 different barcodes (referred to as QuipuNet). In addition, a recent work proposed the S2Snet method (Guan *et al.* 2022), which achieved an accuracy of 0.948 in barcode recognition tasks on the same dataset. We use their reported results for comparison. Furthermore, we expanded our baselines with two additional modern models, the transformer (Vaswani *et al.* 2017) and a self-supervised method developed for nanopore DNA sequencing (Vintimilla and Hwang 2024) referred to as SimCLR.

In comparison, NanoSSL demonstrated the superior performance in barcode recognition tasks (mean ± SD from 5 experiments: accuracy 0.951 ± 0.001, F1 score 0.951 ± 0.001, precision 0.950 ± 0.003, and recall 0.952 ± 0.001). These results showed a modest but consistent increase in performance compared to all prior work across all metrics except for the precision vs. Human (Table 2). These results suggest that NanoSSL uncovers rich feature representations in nanopore data through self-supervised pre-training. NanoSSL showed robust performance across numerous data categories and imbalanced data samples. This consistency may be a result from NanoSSL’s adaptability to different data scales owing to the nanopore event fragmentation process.

**Figure 3.**
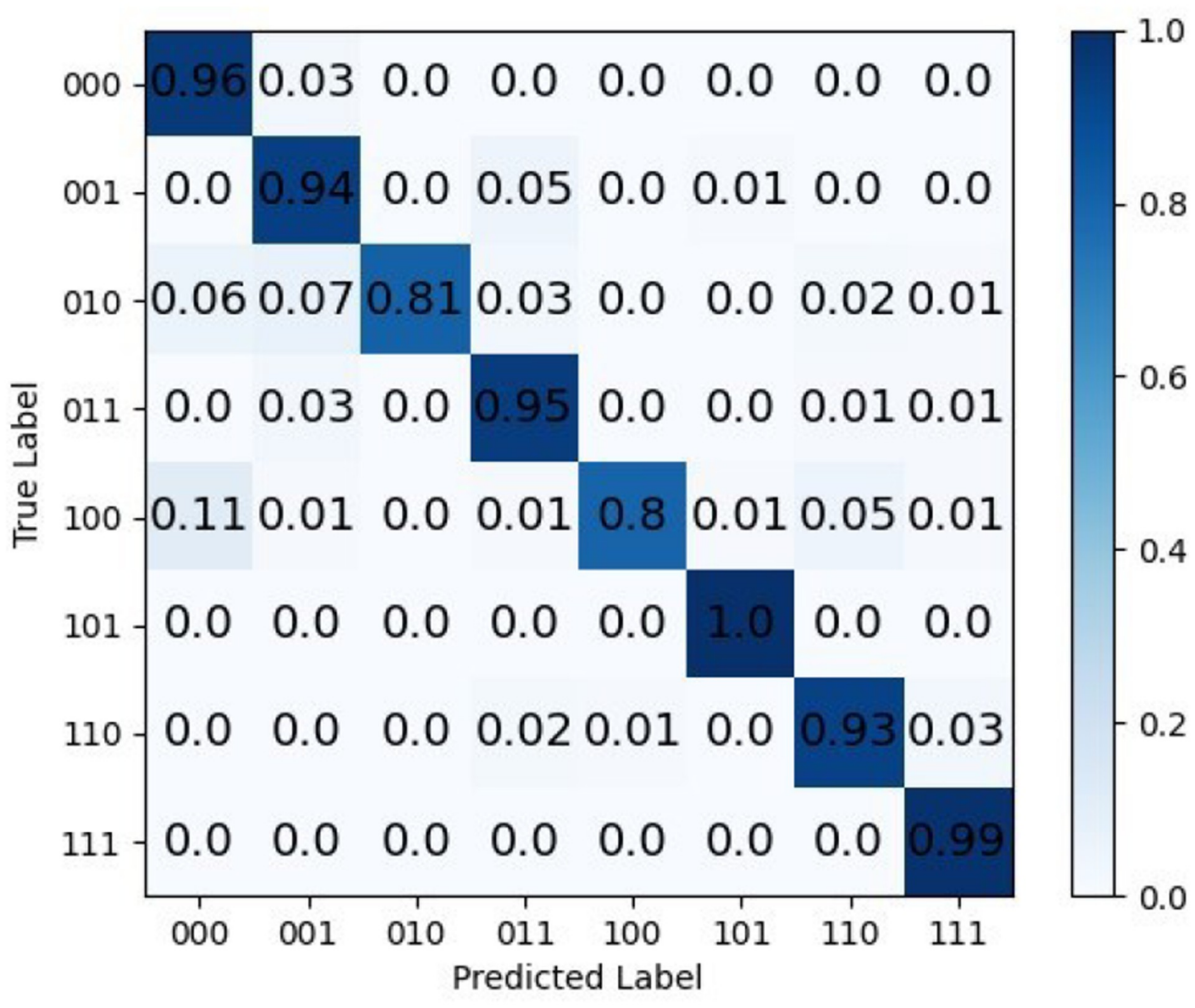
Classification confusion matrix of antigen antibody barcode using NanoSSL.

The confusion matrix for barcode recognition is shown in Fig. 3. NanoSSL classification performance is outstanding for most of barcodes except for the categories “010,” and “100.” This can be attributed to the varying sample sizes among different categories in the training set. For example, the training set of “010” and “100” contains 2319 and 876 events, respectively, and at the same time their test set has 101 and 83 events, respectively. In contrast, each training set in the other 6 barcode categories owns over 5000 events, confirming that a larger sample size is beneficial to improving the experimental results.

#### 3.1.2 Identification of target protein binding

In the ONT multiplexed data, the latter half of the DNA vector was designed to contain a specific binding site and allowed a target protein molecule to bind. The accuracy of identifying whether or not the target protein successfully bound reached 0.971 using QuipuNet. In this study, NanoSSL was used to detect the same targeted proteins, and achieved an accuracy of detecting protein binding of 0.982 ± 0.001. Other metrics scored similarly and consistently under repetition (precision 0.984 ± 0.004, recall 0.969 ± 0.004, F1 score: 0.976 ± 0.002). Overall, NanoSSL outperformed QuipuNet in accuracy, precision, and F1 score, except for a comparable performance in recall (Table 3).

It is worth mentioning that it is necessary for the previous methods to train two separate models to optimize the performance because to analyze the ONT multiplexed nanopore data needs to implement the barcode recognition and the target protein binding recognition, which involves the eight-element classification problem and the binary classification problem in deep learning classification tasks, respectively. However, NanoSSL can initialize a model through pretraining competent enough to carry out such two-step classification task alone with fine-tuning. Not only was the downstream classification performance improved, but the training process was simplified due to the advantages of NanoSSL in feature extraction and generalization ability, as well as efficiency and flexibility in handling multi-task learning scenarios. Importantly, the ability to make use of a single pretrained model across a wide range of nanopore classification tasks is a significant advance in standardization of nanopore analysis.

#### 3.1.3 Visualizing label-free feature selection

In order to understand each process in NanoSSL, including its feature learning ability, t-SNE dimensional reduction (Van Der Maaten and Hinton 2008) was used to visualize the barcode recognition task of the ONT multiplexed protein sensing dataset to intuitively compare the feature distributions output by encoders under different training strategies.

**Figure 4.**
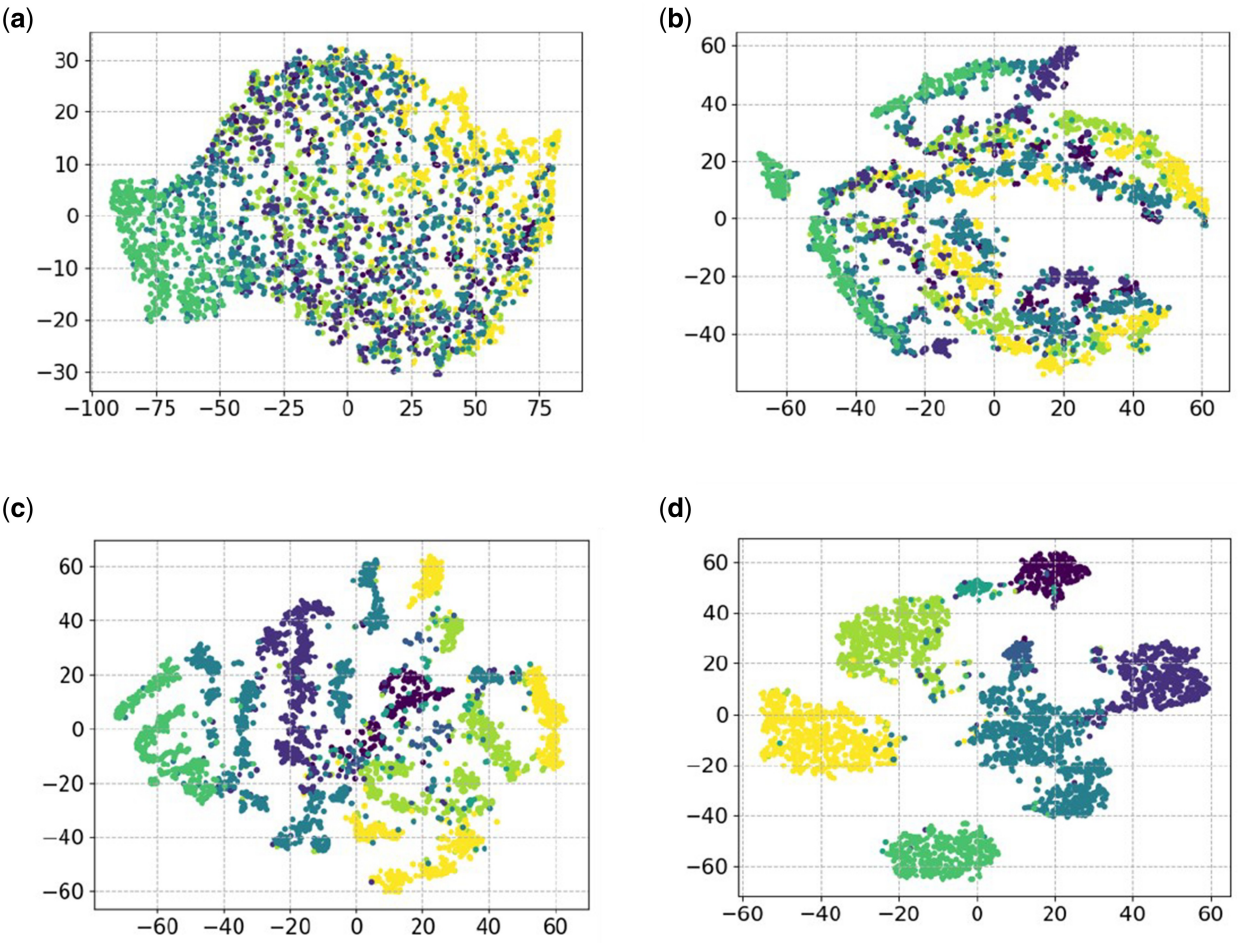
Visualization of NanoSSL encoder’s output using t-SNE at different training stages. (a) Visualization of multiplexes nanopore data features derived from randomly initialized untrained encoders. (b) Feature visualization of pre-trained NanoSSL. (c) and (d) present the visualization results of NanoSSL-ZeroTrain and whole NanoSSL, respectively.

The features derived by the randomly initialized encoder exhibited a relatively scattered distribution in Fig. 4a, where the encoder distinguished different categories without any effective representation learning method. Later, the features extracted by the pretrained model began to concentrate and cluster (Fig. 4b). Gradually, the data points within each category exhibited tight clustering and separation between categories. This visualization shows how the representations from self-supervised pre-training can capture important features of the data without labels. Pre-training the model not only enables the recognition of categories, but also enhances the similarity between similar samples, which is crucial for label-free classification tasks.

Although the discriminative power of features was strengthened through the task-specific supervised training version called NanoSSL-ZeroTrain, which skips the self-supervised pretraining, there still appeared flaws in terms of inter-category separation and intra-category aggregation (Fig. 4c). The outcome from pre-training plus fine-tuning in Fig. 4d maintained the high clustering of the encoder during pretraining as well as led to complete and precise separations among categories. In other words, the features gained during the pre-training phase are beneficial for subsequent supervised learning tasks, and fine-tuning further optimizes these features to adapt to specific classification tasks.

### 3.2 Classification of self-produced nanopore data

#### 3.2.1 Classification of native Aβ_1-42_ from its mutants

In this section, NanoSSL is tested using our nanopore protein sequencing datasets. Specifically, it is desired to distinguish Native Aβ_1-42_ from its two important mutants, E22G and G37R. Other methods to compete with include classical machine learning algorithms adopted in previous studies (Tables 5–9, available as supplementary data at *Bioinformatics* online), transformer, SimCLR, a recent deep learning framework called TandemMod for RNA modification detection in nanopores (Wu *et al.* 2024), as well as NanoSSL-ZeroTrain. The numerals in Table 4 are the average of 5-fold cross validation and indicate that NanoSSL possesses superior performance on the classification between the native and the mutant Aβ_1-42_ proteins. For E22G, the accuracy and F1-score are 0.895 ± 0.015 and 0.887 ± 0.015; while for G37R, they are 0.925 ± 0.003 and 0.945 ± 0.002, respectively. The NanoSSL-ZeroTrain model also participates in the comparison. On the one hand, it provides a comparable performance to the other machine learning methods, which proves that the attention mechanism is helpful. On the other hand, such untrained results can be further improved by the whole version equipped with pretraining and fine-tuning, reflecting the impressive representation learning ability of the self-supervised pretraining strategy. Consequently, the upper limit of model performance in particular for nanopore data analysis is increased.

#### 3.2.2 Performance under data scarcity

**Figure 5.**
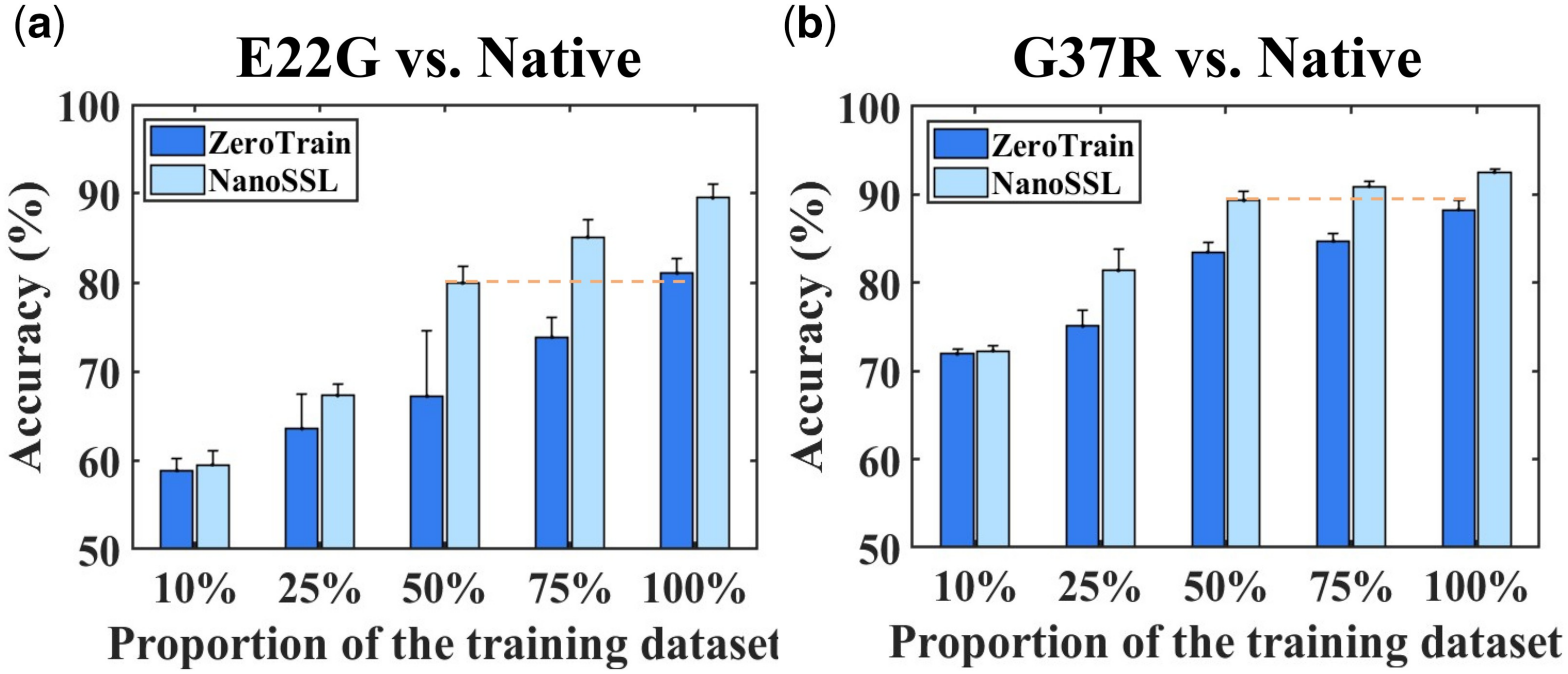
Performance comparison between NanoSSL and NanoSSL-ZeroTrain at various sizes of training set. (a) and (b) presenting the accuracy results regarding the classification of mutants E22G and G37R from Native Aβ_1-42_, respectively.

Self-supervised pretrained models have demonstrated extraordinary accuracy and generalization ability when facing data scarce scenarios (Rafiei *et al.* 2024). In order to explore the ability in dealing with limited nanopore data, the adaptability of NanoSSL was studied by creating sparse data conditions by varying the partitioning of the training sets. Specifically, 10%, 25%, 50%, 75%, and 100% of the original training set were tested for the model adaptability to nanopore data size variation. In addition, the same test set was retained in each condition. The performance under each condition was compared between NanoSSL and NanoSSL-ZeroTrain using five-fold cross validation and shown in Fig. 5. As expected, the performance improves as the training set size increases, especially when the size is small (10%–50%). Additionally, NanoSSL constantly performs better than NanoSSL-ZeroTrain on the classification of both E22G and G37R. When 50% training set was used, NanoSSL already reached a comparable or superior classification performance to NanoSSL-ZeroTrain with 100% training set. This highlights that to some extent NanoSSL can solve the problem of data scarce because NanoSSL is forced to learn abundant representations of nanopore signals by constructing unobserved segments of the predicted data. At last, more useful features are extracted to implement the subsequent classification.

#### 3.2.3 Ablation experiment

**Figure 6.**
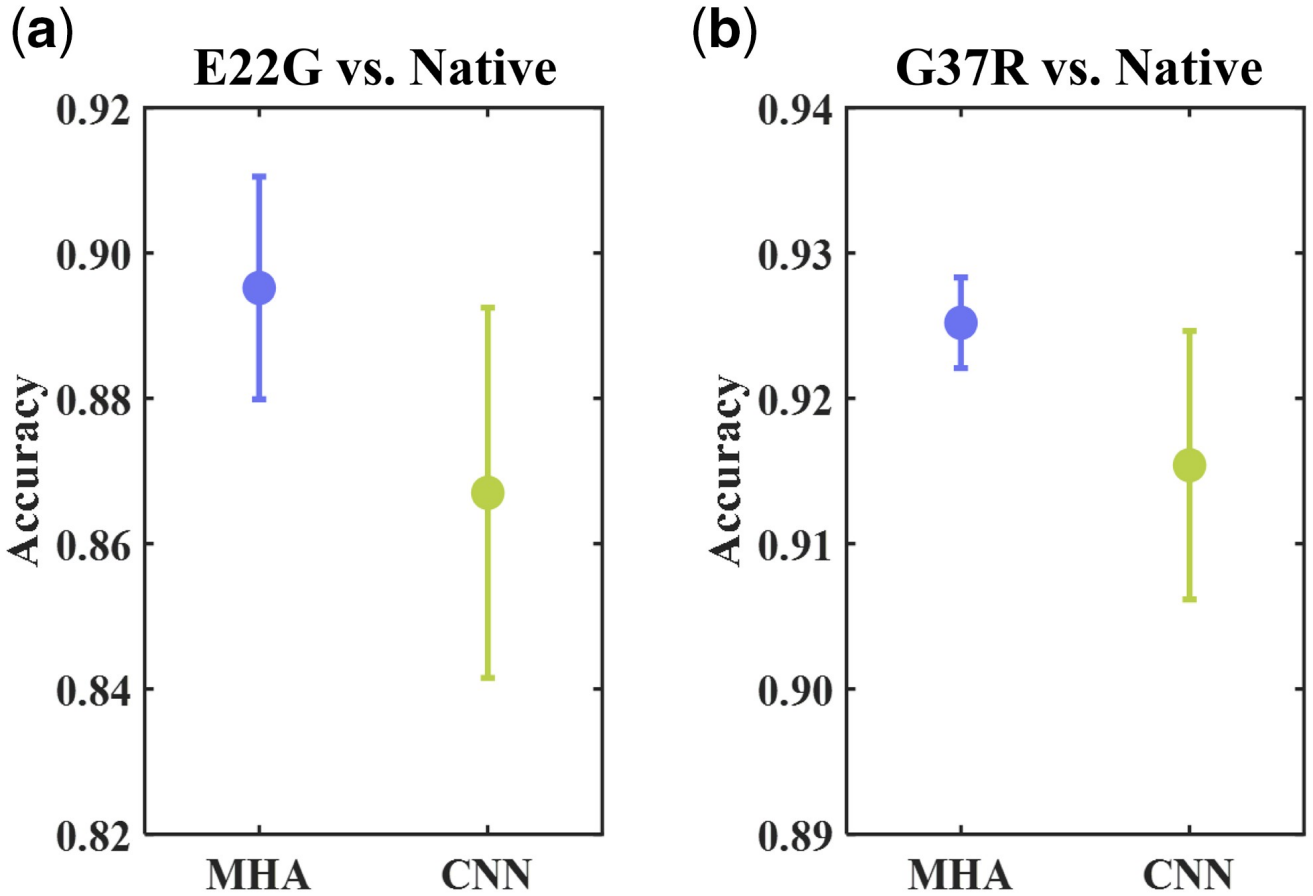
Performance comparison of protein classification between multi-head attention mechanism and CNNs in NanoSSL. (a) and (b) The accuracies in 5-fold cross validation for mutants E22G and G37R classification, respectively. MHA and CNN stand for multi-head attention and CNNs, respectively.

An ablation experiment was conducted to learn the role that certain key components played in NanoSSL. In ablation, each layer in the multi-head attention mechanism was replaced with CNNs to compare the performance on recognizing the same mutants. Figure 6 illustrates that the model equipped with a multi-head attention mechanism surpassed the CNN equivalents in classifying both mutants under five-fold cross validation. Unlike the local receptive field of CNNs, the attention mechanism owns the global perception ability and, therefore, appears to be more competent for handling nanopore tasks. Furthermore, it is inferred that the multi-head attention mechanism is more robust according to the standard deviation in accuracy.

#### 3.2.4 Tuning the masking ratio

**Figure 7.**
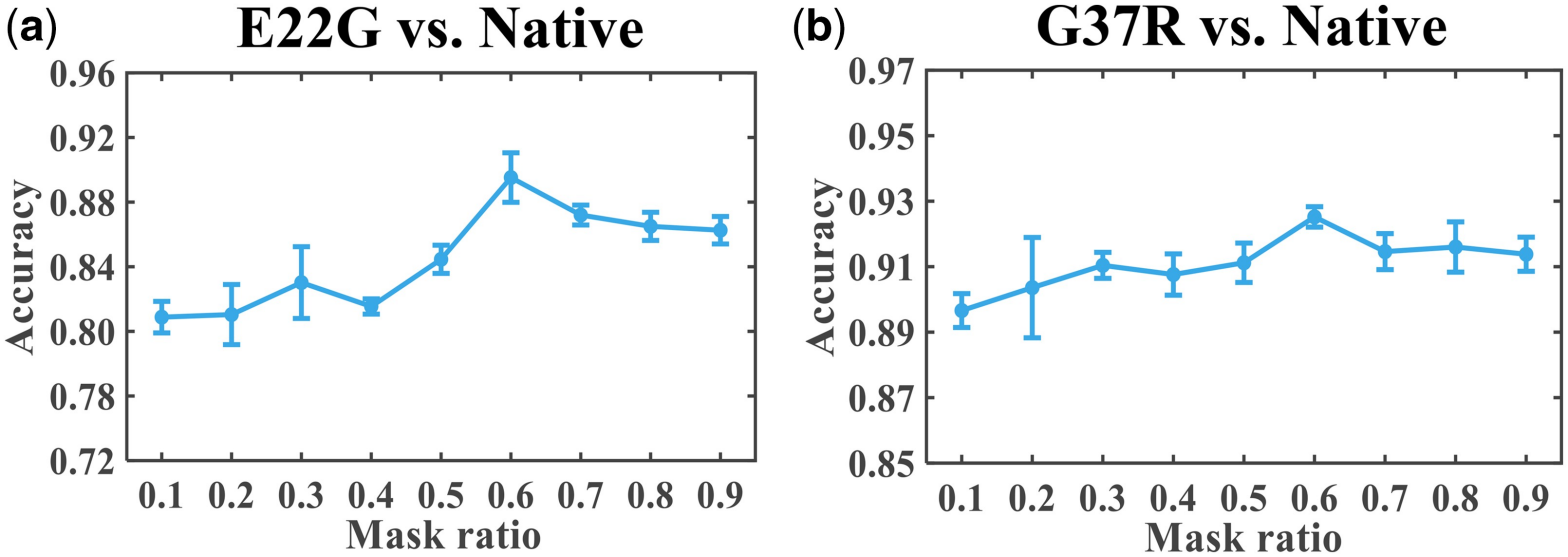
Mask ratio dependency of NanoSSL under 5-fold cross validation. (a) and (b) track the accuracies at varied mask ratios for the classification of E22G and G37R, respectively.

An important hyperparameter in NanoSSL during self-supervised phase is the mask ratio r. It determines the proportion of the input sequence X that needs to be masked before reconstruction during pre-training. Figure 7 demonstrates that the classification accuracy reached the apex when r was set to be 0.6 for both mutated Aβ_1-42_. In the field of generative self-supervised learning in natural language processing, lower mask ratios, e.g. 0.15, are generally conducive to better results (Devlin *et al.* 2019); while in the field of generative self-supervised learning in image processing, higher mask ratios, e.g. 0.75, are preferred (He *et al.* 2022). Due to the lower information density of single nanopore signal compared to natural language, a lower mask ratio may result in a simplicity bias during pretraining and a lack of the requisite complexity to foster robust feature representations with strong generalization capability. On the contrary, a higher mask ratio would drive NanoSSL to perform more difficult reconstruction tasks, force the encoder to dig deeper into each nanopore signal to reconstruct proper masked positions, and thereby promote the collection of sufficient universal feature representations. However, an excessively high mask ratio could make the task too difficult. In short, the mask ratio is crucial during pre-training, and the risk of overfitting can be alleviated by setting this hyperparameter to an appropriate value.

#### 3.2.5 Classification of Aβ_1-42_ with chemical modifications

Together with the efforts on protein mutation identification in nanopore data using deep learning methods (Cao *et al.* 2024), pre-trained models of self-supervised learning have been extended to other important tasks, such as detecting chemical modifications (Danilevsky *et al.* 2022, Li *et al.* 2023). NanoSSL was also tested to classify Aβ_1-42_ variants with post-translational modifications (PTMs). For example, to classify phosphoserine at position 26 (denoted as S26PO_4_) yielded an average classification accuracy of 0.786 (Section 6, available as supplementary data at *Bioinformatics* online). It is speculated that, unlike mutations, chemical modifications do not directly change the primary structure, even though PTMs could cause protein conformational variations due to charge, hydrophobicity, etc. Besides that, phosphorylation leads a local volume increase by 0.0565 nm^3^ according to crystallography data, while E22G and G37R cause a local volume change of 0.0887 nm^3^ and 0.1357 nm^3^, respectively. It was found that along unfolded polypeptide chains, the larger the volume variation due to PTM (or mutation), the stronger the nanopore localized signal (Chen *et al.* 2024). Currently, it is still challenging for NanoSSL to persuasively identify chemical modifications at this resolution. Encouragingly, large language models have demonstrated an impressive ability to handle complex sequences by combining large-scale datasets with large parameter models, and there may be similar gains in sequence recognition and prediction for nanopores given sufficiently large and representative training datasets. In addition, it is promising to consider the integration of diverse nanopore datasets, such as data from solid-state nanopores and biological nanopores across various detection categories, or the use of attention mechanisms to construct deep learning models with more parameters or novel architectures (Ahsan *et al.* 2024). Overall, applying NanoSSL to a broader set of proteomic datasets may yield benefits.

## 4 Conclusion

This study proposes a deep learning method, NanoSSL, that is capable of handling diverse classification tasks across different nanopore protein data sources and analytes. The framework is designed for broad protein identification tasks by capturing as many features as possible in individual nanopore ionic current temporal signals leveraging recent advances in deep learning. After learning the general feature representations during the self-supervised pre-training stage in each specific protein recognition task, a consistent increase in classification performance was achieved on public nanopore multiplexed data by replacing traditional neural networks with a multi-head attention mechanism. Furthermore, the flexibility and scalability of NanoSSL is enhanced by adopting a modular design consisting of multiple components, which makes NanoSSL highly adaptive to diverse tasks, thereby addressing a key limitation of current algorithms. The performance of NanoSSL classification of unlabeled mutated Aβ_1-42_ single molecules also exceeds existing analysis methods. The efficiency in handling multi-task learning scenarios and the competence when facing data scarce problems implies that NanoSSL is well-suited to the demands of next-generation proteomic applications of nanopores.

## Supplementary Material

btaf657_Supplementary_Data

## Data Availability

Self-produced solid-state nanopore protein sequencing data is available by request to the corresponding author.
