# Supplementary material for: NanoSSL: attention mechanism-based self-supervised learning method for protein identification using nanopores
Source: Bioinformatics. 2025 Dec 5;42(1):btaf657. doi: 10.1093/bioinformatics/btaf657 (PMC12777981; doi:10.1093/bioinformatics/btaf657)
Supplement: btaf657_Supplementary_Data [file btaf657_supplementary_data.docx]

Supplementary Information

NanoSSL: attention mechanism-based self-supervised learning method for protein identification using nanopores

Yong Xie^1^, Jindong Li^1^, Ziyan Zhang^1^, Bin Meng^1^, Shuaijian Dai^1^, Yuchen Zhou^2^, Eamonn Kennedy^3^, Niandong Jiao^4^, Haobin Chen^1,5,6^, Zhuxin Dong^1,5,6,*^

^1^Department of Biomedical Engineering, Xiangya School of Basic Medical Sciences, Central South University, Changsha, Hunan 410013, China

^2^Xiangya School of Medicine, Central South University, Changsha, Hunan 410013, China

^3^Division of Epideminology, Internal Medicine, University of Utah, Salt Lake City, UT 84112, USA

^4^State Key Laboratory of Robotics, Shenyang Institute of Automation, Chinese Academy of Sciences, Shenyang, Liaoning 110169, China

^5^Furong Laboratory, Changsha, Hunan 410000, China

^6^National Engineering Research Center of Personalized Diagnostic and Therapeutic, Changsha, Hunan 410000, China

*Corresponding author, email: [dongzhuxin@csu.edu.cn](mailto:dongzhuxin@csu.edu.cn)

## 1. Training efficiency

To specify the performance of the subsequence segmentation mechanism and the encoder’s separate processing of the visible and masked segments on the improvement of pre-training efficiency, ablation experiments are conducted with ONT dataset with antigen-antibody barcode identification task. The results from ablation experiments are listed in **Table S1**.

**Table S1.** The indication of training efficiency improvement by ablation experiments

| Label | Training cost per epoch |
| --- | --- |
| NanoSSL | 21 s |
| w/o parallel processing | 31 s |
| w/o subsequence segmentation | 216 s |

## 2. Dataset detail

**Table S2.** Number of barcode events in the ONT dataset

| Label | Training samples | Test samples |
| --- | --- | --- |
| 000 | 5593 | 253 |
| 001 | 8155 | 502 |
| 010 | 2319 | 101 |
| 011 | 15178 | 827 |
| 100 | 876 | 83 |
| 101 | 7251 | 427 |
| 110 | 6473 | 606 |
| 111 | 6680 | 665 |

**Table S3.** Number of target protein binding events in the ONT dataset

| Label | Training samples | Test samples |
| --- | --- | --- |
| unbound | 36551 | 2191 |
| bound | 15874 | 1273 |

**Table S4.** Amino acid sequences of native and mutated Aβ_1-42_

| Protein | Sequence |
| --- | --- |
| Native | DAEFRHDSGYEVHHQKLVFFAEDVGSNKGAIIGLMVGGVVIA |
| E22G | DAEFRHDSGYEVHHQKLVFFA**G**DVGSNKGAIIGLMVGGVVIA |
| G37R | DAEFRHDSGYEVHHQKLVFFAEDVGSNKGAIIGLMV**R**GVVIA |

## 3. NanoSSL framework Detail

### 3.1 Signal projection module

Before inputting into the encoder, it is necessary to use the input projection module to linearly map the sub serialized nanopore data $P$ to the d-dimension $d$：

$$\begin{aligned} P'=\mathrm{Proj}\left( P \right)=P*W+b \end{aligned}$$

where the weight matrix $W\in\mathbb{R}^{T/L\times d}$，the bias vector $b\in\mathbb{R}^{d\times1}$。The input $X$ of the encoder is the nanopore data $P'\in\mathbb{R}^{L\times d}$ after linear mapping, plus the learnable position embedding $PE\in\mathbb{R}^{L\times d}$：

$$\begin{aligned} X=P^{'}+PE \end{aligned}$$

A masking strategy is used to select random positions in the input sequence *X* in length *L* for masking, which means the number of masked positions is *L*×*r*, where *r* is the mask ratio. *X* is then split into the masked portion, *X_m_*, and the original visible portion, *X_v_*.

### 3.2 Multi-head attention mechanism

The encoder consists of multiple layers of cascaded multi-head self-attention modules that learn the contextual representation of nanopore data input. In the attention module, the input matrix $X$is first linearly mapped into three different matrices $Q, K, V \in\mathbb{R}^{L\times d}$, denoted as query, key, and value. The goal is to map each query and a series of key value pairs to the output. The implementation of attention mechanism can be expressed as the following:

$$\begin{aligned} \text{Attention }\left( Q, K, V \right)=\mathrm{softmax} \left( \frac{QK^{T}}{\sqrt{d_{k}}} \right)V \end{aligned}$$

where $d_{k}$ in the scaling factor $\frac{1}{\sqrt{d_{k}}}$ is the dimension of key，$\mathrm{softmax}(z_{i})=\frac{e^{z_{i}}}{\sum_{j=1}^{d_{k}} e^{z_{j}}}$ converts attention scores into probability values ranging from [0,1].

In the multi-head attention module, $h$ independent and parallel linear transformations are used to map queries and keys to the $d_{k}$ dimension, and values to the $d_{v}$ dimension, which can help the model focus on potential information in different feature spaces. $h$ is the number of attention heads. Parallel execution of $h$ attention functions, followed by connecting the outputs of these attention heads.

$$\text{MHA}\left( Q,K, V \right)=concat\left( {head}_{1},{head}_{2},\ldots,{head}_{h} \right),$$

$$\begin{aligned} {head}_{i} = \text{Attention }\left( Q_{i}, V_{i}, K_{i} \right) \end{aligned}$$

where $Q_{i}\in\mathbb{R}^{L\times d_{k}}, K_{i}\in\mathbb{R}^{L\times d_{k}}, V_{i}\in\mathbb{R}^{L\times d_{v}}$. In this study, the encoder adopts self-attention mechanism, while the decoder adopts cross-attention mechanism (**Fig. S1**).

Each multi-head attention module includes a Feed Forward Network (FFN). The architecture of FFN consists of two linear layers, with a ReLU and a Dropout between the two fully connected layers:

$$\begin{aligned} \mathrm{FFN}\left( x \right)=\left( \max\left( 0\mathbf{,}\left( xW_{1}+b_{1} \right)\cdot dropout\left( p_{1} \right) \right)W_{2}+b_{2} \right) \end{aligned}$$

where $x$ is the input of FFN，$W_{1}\in\mathbb{R}^{d\times4d}and W_{2}\in\mathbb{R}^{4d\times d}$ are weight matrices，$b_{1}\in\mathbb{R}^{4d\times1}and b_{2}\in\mathbb{R}^{d\times1}$ are bias vectors，max(0, *z*) represents the ReLU activation function, and $dropout(p_{1})$ represents Dropout with a rate of $p_{1}$. Attention and FFN are followed by layer normalization and residual connections.

**Figure S1. Two types of attention mechanisms.** The left represents the self-attention mechanism, where query, key, and value come from the same sequence. The right represents the cross-attention mechanism, where query (elements in the red box) comes from one sequence, while key and value are from another sequence.

### 3.3 Encoder

This study adopts an asymmetric encoder, where the masked parts $X_{m}$ and the visible parts $X_{v}$ are two separate inputs for bidirectional encoding while they share the weights of the encoder (**Fig. S2**). This architecture brings two significant advantages: one is the reduction of computational burden for the encoder. Since the encoder only needs to focus on either visible or masked subsequences, the information for processing is reduced. As a result, the computational complexity of the attention mechanism for individual nanopore signals is reduced. The other is the similarity between pre-training and fine-tuning. The whole sequence of every nanopore signal is the input during fine-tuning, so there is no need for masking operation. Therefore, processing the visible and masked parts separately during pre-training helps to maintain a satisfactory consistency in the input data with fine-tuning. The output vector of the last layer of the encoder (8 layers in total by default) is calculated by $H^{8}=H_{v}^{8}+ H_{m}^{8}= \left. \{h_{1}^{8},h_{2}^{8} , ...,h_{L}^{8} \} \right.\in\mathbb{R}^{L\times d}$. It gives the global contextual representation of the nanopore signals learned by the encoder, where $h_{i}$ represents the output of the encoder at position $i$.

**Figure S2. The encoder framework.**

### 3.4 Decoder

The decoder consists of 8 layers of multi-head cross-attention modules and aims to reconstruct the feature representation of the masked part through the visible part of each nanopore signal (**Fig. S3**). In this study, the query in cross-attention is the masked part in a nanopore temporal signal, and the query’s keys and values are estimated from the visible portion. This mechanism enables the model to focus on the mask parts at each layer. The output of cross-attention mechanism is a new representation of the masked part. Firstly, the decoder extracts the visible part $H_{v}^{8}$ from the output vector $H^{8}$ of the encoder, and replaces the masked vector $H_{m}^{8}$ at each position with a randomly initialized vector $x_{m} \in\mathbb{R}^{d}$. Then, $x_{m}$ and $H_{v}^{8}$ are overlapped while keeping the encoded positions the same as the original subsequence. In the cross-attention mechanism, elements in the masked positions are considered as queries, while the other elements are considered as keys and values. The decoder updates its representation by weighting the keys and values to the masked positions. The output vector for the masked position of the last layer (default 8 layers) of the decoder can be described as $F_{m}^{8}= \left. \{f_{1}^{8},f_{2}^{8} , ...,f_{L*r}^{8} \} \right.$, where $f_{i}$ represents the output of the decoder at position $i$.

**Figure S3. The decoder framework.** The input consists of visible parts and randomly initialized masked parts (marked as **M**), which is then decoded to output a prediction for the masked parts (marked as **P**).

### 3.5 Classifier

The classifier in NanoSSL consists of two fully connected layers. The first layer aims to extend the input to a higher dimensional feature space, $d_{c}$, to reveal complex correlations in the data, and introduce nonlinear properties through ReLU activation function and Dropout for the generalization ability enhancement. The second layer is responsible for mapping these high-dimensional features onto the number of pre-defined categories and outputting the original scores $\hat{y}_{c}$ for each category, which can be described as following:

$$\mathrm{classifier}\left( z \right)=\max\left( 0\mathbf{,}\left( zW_{3}+b_{3} \right)\cdot dropout\left( p_{2} \right) \right)W_{4}+b_{4}$$

where *z* is the input to the classifier, $W_{3}\in\mathbb{R}^{d\times d_{c}}and W_{4}\in\mathbb{R}^{d_{c}\times M}$ are the weight matrices, $b_{3}\in\mathbb{R}^{d_{c}\times1}and b_{4}\in\mathbb{R}^{M\times1}$ are the bias vectors, max (0, *z*) represents the ReLU activation function, and $dropout(p_{2})$ represents the Dropout with a rate of $p_{2}$.

## 4. Metric Computation

In this study, the classification results of nanopore data can be described in detail using a confusion matrix. The confusion matrix displays the relationship between the actual labels and the predicted labels for each category, where rows represent the actual categories and columns represent the predicted categories. The confusion matrix has four components: True Positive (TP) is the number of correctly classified positive samples; False Positive (FP) is the number of misclassified negative samples; True Negative (TN) is the number of correctly classified negative samples; False Negative (FN) is the number of misclassified positive samples. Therefore, several critical indicators for performance assessment can be calculated by this matrix. Accuracy (ACC) is the most intuitive performance metric, representing the proportion of correctly classified samples to the total sample. The formula to calculate ACC is defines as:

$$ACC=\frac{TP+TN}{TP+TN+FP+FN}$$

F1 Score is the harmonic average of Precision and Recall, where Precision refers to the proportion of samples predicted as positive that are actually positive, and Recall refers to the proportion of actual positive samples that are correctly predicted as positive.

$$\begin{aligned} Precision=\frac{\mathrm{TP}}{TP+FP} \end{aligned}$$

$$\begin{aligned} R\mathrm{ecall}=\frac{\mathrm{TP}}{TP+FN} \end{aligned}$$

It is worth noting that when calculating accuracy and recall in multiclass classification problems, one category is treated as a positive class while the other categories are treated as negative classes at a time. Then, accuracy and recall are calculated separately for each category. The formula for calculating F1 score in binary classification problems can be described as follows:

$$F1 score=2*\frac{Precision*Recall}{Precision+Recall}$$

while the formula in multivariate classification problems is:

$$F1 score=\frac{1}{M}\sum_{C=1}^{M} 2*\frac{\mathrm{Precision}_{c}*\mathrm{Recall}_{c}}{\mathrm{Precision}_{c}+\mathrm{Recall}_{c}}$$

where *M* is the total number of categories.

## 5. Parameters in other methods for NanoSSL to compete with

**Table S5.** Hyperparameter of SVM

| Hyperparameter | Value |
| --- | --- |
| C | 1.0 |
| gamma | ’scale’ |
| kernel | ’rbf’ |

**Table S6.** Hyperparameter of RF

| Hyperparameter | Value |
| --- | --- |
| n_estimators | 300 |
| max_depth | 10 |
| criterion | ’entropy’ |
| max_features | 'auto' |
| min_samples_leaf | 1 |

**Table S7.** Hyperparameter of XGBoost

| Hyperparameter | Value |
| --- | --- |
| n_estimators | 100 |
| max_depth | 10 |
| learning_rate | 0.1 |
| min_child_weight | 1 |

**Table S8.** Hyperparameter of CNN

| Hyperparameter | Value |
| --- | --- |
| out_channels | 64 |
| kernel_size | 3 |
| stride | 1 |
| padding | 1 |

**Table S9.** Hyperparameter of LSTM

| Hyperparameter | Value |
| --- | --- |
| hidden_size | 64 |
| num_layers | 8 |
| batch_first | True |

##

## 6. PTM detection using NanoSSL

An attempt to classify Native Aβ_1-42_ and S26PO_4_ Aβ_1-42_ gives an average classification accuracy of 0.786, and the confusion matrix for such classification is shown in **Fig. S4**.


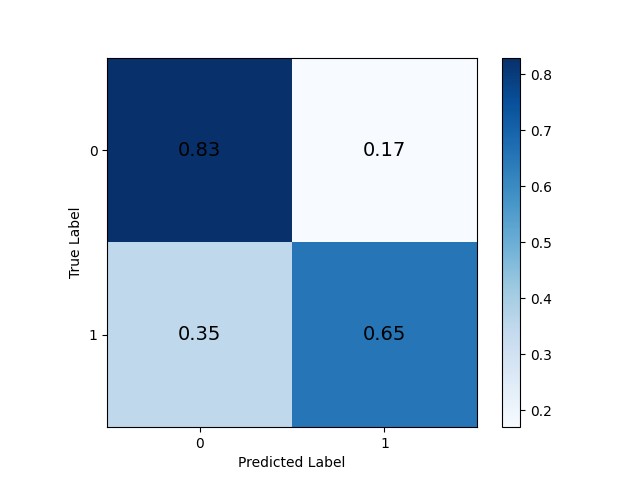


**Figure S4. Classification confusion matrix between native and phosphoserine Aβ_1-42_.**
